# Causal associations between liver traits and Colorectal cancer: a Mendelian randomization study

**DOI:** 10.1186/s12920-023-01755-w

**Published:** 2023-12-06

**Authors:** Ying Ni, Wenkai Wang, Yongming Liu, Yun Jiang

**Affiliations:** 1 Beijing Normal University, 100875 Beijing, China; 2 Department of Oncology, Shuguang Hospital, Shanghai University of Traditional Chinese Medicine, 200021 Shanghai, China; 3 Shi’s Center of Orthopedics and Traumatology, Shuguang Hospital, Shanghai University of Traditional Chinese Medicine, 200021 Shanghai, China; 4 Institute of Traumatology & Orthopedics, Shanghai Academy of Traditional Chinese Medicine, 200021 Shanghai, China

**Keywords:** Mendelian randomization, Colorectal cancer, Liver traits, Colorectal liver Metastasis

## Abstract

**Objective:**

This study aimed to investigate the causal associations between several liver traits (liver iron content, percent liver fat, alanine transaminase levels, and liver volume) and colorectal cancer (CRC) risk using a Mendelian randomization (MR) approach to improve our understanding of the disease and its management.

**Methods:**

Genetic variants were used as instrumental variables, extracted from genome-wide association studies (GWAS) datasets of liver traits and CRC. The Two-Sample MR package in R was used to conduct inverse variance weighted (IVW), MR Egger, Maximum likelihood, Weighted median, and Inverse variance weighted (multiplicative random effects) MR approaches to generate overall estimates of the effect. MR analysis was conducted with Benjamini-Hochberg method-corrected P values to account for multiple testing (P < 0.013). MR-PRESSO was used to identify and remove outlier genetic variants in Mendelian randomization (MR) analysis. The MR Steiger test was used to assess the validity of the assumption that exposure causes outcomes. Leave-one-out validation, pleiotropy, and heterogeneity testing were also conducted to ensure the reliability of the results. Multivariable MR was utilized for validation of our findings using the IVW method while also adjusting for potential confounding or pleiotropy bias.

**Results:**

The MR analysis suggested a causal effect between liver volume and a reduced risk of CRC (OR 0.60; 95% CI, 0.44–0.82; P = 0.0010) but did not provide evidence for causal effects of liver iron content, percent liver fat, or liver alanine transaminase levels. The MR-PRESSO method did not identify any outliers, and the MR Steiger test confirmed that the causal direction of the analysis results was correct in the Mendelian randomization analysis. MR results were consistent with heterogeneity and pleiotropy analyses, and leave-one-out analysis demonstrated the overall values obtained were consistent with estimates obtained when all available SNPs were included in the analysis. Multivariable MR was utilized for validation of our findings using the IVW method while also adjusting for potential confounding or pleiotropy bias.

**Conclusion:**

The study provides tentative evidence for a causal role of liver volume in CRC, while genetically predicted levels of liver iron content, percent liver fat, and liver alanine transaminase levels were not associated with CRC risk. The findings may inform the development of targeted therapeutic interventions for colorectal liver metastasis (CRLM) patients, and the study highlights the importance of MR as a powerful epidemiological tool for investigating causal associations between exposures and outcomes.

**Supplementary Information:**

The online version contains supplementary material available at 10.1186/s12920-023-01755-w.

## Introduction

Colorectal cancer(CRC) is the third most common type of cancer in the world and more than 50% of patients with CRC will develop colorectal liver metastases (CRLMs) [1, 2]. The liver is the most common site of colorectal metastasis, with 15–20% of patients being candidates for hepatectomy [3, 4]. Approximately one-third of patients will develop liver metastases within three years of diagnosis, which contributes to the overall poor prognosis and survival rates [5]. Surgical resection is currently the treatment of choice for colorectal liver metastasis (CRLM) and has been shown to be the only potentially curative therapy [6, 7]. The combination of advances in medical therapy, such as systemic chemotherapy (CTX), and the role of surgery in the treatment of metastatic disease has had a positive impact on the prognosis, with an increase in median survival and cure rates [8]. However, the biology of liver metastases is extremely complex and involves many interactions between tumor cells and the liver microenvironment, leading to significant challenges in the management of CRLM [9]. Consequently, there is an urgent need to explore novel therapeutic strategies and elucidate the underlying mechanisms linking liver traits to CRC development and progression.

The liver plays a crucial role in the natural course of CRC due to its anatomical and physiological importance. Metastatic cells in CRC acquire properties in addition to those required to become neoplastic because they are able to successfully dissociate, disseminate and colonize secondary sites: motility and invasion, the ability to modulate the secondary site or the local micro-environment, plasticity and the ability to colonize secondary tissues [10, 11]. In addition to the mucus and intestinal epithelial barrier, the intestine also has an intestinal vascular barrier (GVB), which acts as a gatekeeper to control the entry of molecules and microorganisms into the systemic circulation [12, 13]. Under hazardous conditions, harmful intestinal pathogens can cross the epithelial barrier, damaging the GVB and ultimately allowing the bacteria or their components to spread into the bloodstream, eventually reaching the liver [12, 13].

The liver and lung are the most common sites for CRC metastasis, with the liver’s predominant blood supply arising from the confluence of the gastrointestinal (GI) tract, which supplies blood vessels via the hepatic portal vein. For metastases arising from the colon and proximal parts of the rectum, the portal system directs blood flow directly to the liver [14]. In hematogenous dissemination, platelets and neutrophils protect circulating tumour cells (CTCs) from being eliminated by NK cells. Subsequent entry of CTCs into the liver microvasculature may trigger a proinflammatory cascade that induces the secretion of chemokines by Kupffer and stellate cells and upregulation of vascular adhesion receptors, resulting in the adhesion of CTCs to the liver microvasculature [15]. Furthermore, tumour cell interaction with the tumour microenvironment (TME) plays an important role in CRLM [16]. By remodeling the TME, cancer cells can induce the formation of pre-metastatic niches [17, 18]. For example, activation of hepatic stellate cells (α-HSC) is the most common biological process in secondary or primary liver cancer [19]. By remodeling and depositing extracellular matrix (ECM), α-HSCs can influence CRC cell growth and invasion [20].

**Fig. 1:**
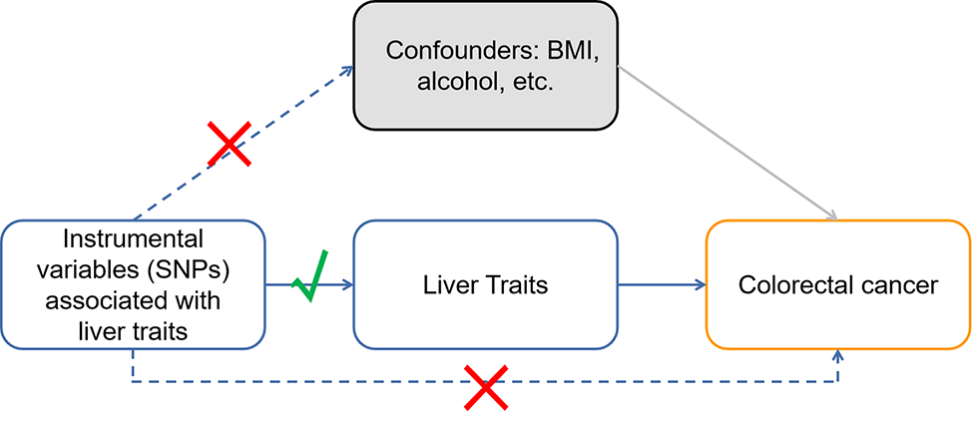
Study design of Mendelian randomization between liver traits and CRC. The solid lines represent the association between the instrumental variables and exposure as well as the association between exposure and outcome. Dash lines with a cross means that the association meets two basic assumptions of Mendelian randomization: (i) the genetic variants are independent of confounders between exposure and outcomes, (ii) the genetic variants only influence the outcome via exposure

Mendelian randomization (MR) is a powerful epidemiological approach that leverages genetic variants as instrumental variables to investigate causal associations between exposures and outcomes [21]. By capitalizing on the random allocation of genetic variants during meiosis, MR studies can provide robust evidence for causality, while minimizing the influence of potential confounding factors and reverse causation [22, 23]. Based on this, in the context of CRC and liver traits, MR presents a unique opportunity to untangle the intricate relationship between liver characteristics and colorectal cancer risk, which could, in turn, shed light on the biological mechanisms underpinning CRLM and inform the development of targeted therapeutic interventions, as depicted in Fig. 1.

This MR study aims to investigate the causal associations between several liver traits (liver iron content, percent liver fat, alanine transaminase levels and liver volume) and CRC risk, with the ultimate goal of improving our understanding of the disease and its management. For this aim, we established an effective and reliable data processing framework, which made it possible to generate instrumental variables (IVs) for MR analysis. By selecting exposure-related genetic variations as IVs, MR eliminates confounding factors [24]. And then, using an inverse variance weighted MR approach, we calculated the pool result based on IVs. To further guarantee the reliability of our method, MR-Egger and Weighted median analysis and leave-one-out validation were conducted in this step too. MR-Egger is able to detect some violations of the standard assumptions of the instrumental variables and provide an estimate of the effect that is not subject to these violations. The weighted median analysis is used to combine data from multiple genetic variants into a single causal estimate [25]. Moreover, pleiotropy and heterogeneity testing were used to minimize false-positive conclusions and the risk of producing unreliable results [26]. What’s more, multivariable MR was utilized for validation of our findings using the IVW method, while also adjusting for potential confounding or pleiotropy bias. Finally, a full report was generated to provide an overview of the results of our study.

## Materials and methods

### Genetic instruments

Summary-level data from the GWAS dataset was fundamental for MR analysis. IVs were extracted from several liver traits (liver iron content, percent liver fat, alanine transaminase levels and liver volume) related GWAS datasets, and CRC related GWAS datasets were then used in the further analysis. To meet the MR assumptions and reduce the bias, the summarized GWAS data was processed. Subsequently, MR analysis involving an inverse variance weighted MR approach, leave-one-out validation, and MR-Egger analysis was used to comprehensively assess the causal effect of several liver traits on the risk of the development of CRC.

### Data sources

We used data from the UK Biobank for the study of liver iron content, percent liver fat, alanine transaminase levels and liver volume [27]. The UK Biobank has approval from the North West Multicenter Research Ethics Committee (https://www.ukbiobank.ac.uk/ethics/), and these ethics regulations cover the work in this study. Summary statistics data for CRC were available from a genome-wide association meta-analysis of 177,028 European-descent individuals (3,022 cases and 174,006 noncases) from FinnGen [28]. A summary of the characterization and covariates adjusted for the datasets employed in this study can be found in the supplementary information (Table S1).

Our study analyzed the summarized data from genome-wide association studies (GWAS) to create instrumental variables (IVs) for MR analysis. In this context, IVs are genetic variants [29]. [30] The criteria we followed for selecting these instrumental variables are as follows: (1) Each individual SNP must show a significant correlation with several liver traits (liver iron content, percent liver fat, alanine transaminase levels, and liver volume) across the entire genomic region, with a significance threshold of P < 5.0 × 10^− 8^. (2) We assessed the linkage disequilibrium (LD) among SNPs through the OpenGWAS API, which contains LD reference panels for the five super-populations in the 1000 Genomes reference panel. The reference panel includes only bi-allelic SNPs with a minor allele frequency (MAF) > 0.010. The entire process utilizes the PLINK clumping method, where SNPs in linkage disequilibrium (LD) within a specific window are pruned. The SNP with the lowest p-value is kept. In this study, only those SNPs that produced the most significant results, among those with an R2 < 0.0010 within a clumping window of 10,000 kb, were kept. 7 SNPs were utilized as instrumental variables for liver iron content, 10 SNPs for percent liver fat, 228 SNPs for liver volume, and 10 SNPs for liver alanine transaminase levels. All instrumental variables in the supplementary information.

### Two-sample mendelian randomization

Using genetic variants as instrumental variables, MR is a technique that is gaining popularity for determining the causal relationships between exposures and outcomes. In order to guarantee the accuracy of causal reasoning, MR is predicated on a number of essential premises. Assumption 1: There is a significant association between the genetic variation used as an instrumental variable and the relevant exposure. This premise is crucial because it guarantees that the genetic variation is a reliable substitute for the exposure, enabling the estimation of a causal impact. Estimates of the causal impact may be skewed if this assumption is broken. Assumption 2: The genetic variation is not linked to any confounding elements that might skew the relationship between exposure and result. The second fundamental tenet of MR is that there are no confounding variables linked with the genetic variant used as an instrumental variable that might skew the relationship between exposure and outcome. Assumption 3: The genetic variant has no other impact on the outcome besides how it affects the exposure [31]. The genetic variant used as an instrumental variable only influences the result through its impact on the exposure, and not through any other pathway, according to the third fundamental tenet of MR.

In this study, the Two-Sample MR package (version 0.5.6) in R 4.1.3 (R Foundation for Statistical Computing, Vienna, Austria) was used to conduct MR analysis, evaluating the causal effect of various liver traits (including liver iron content, percent liver fat, alanine transaminase levels, and liver volume) on CRC risk mediated by each individual instrumental SNP. Five methods, including inverse variance-weighted (IVW), MR-Egger, Maximum likelihood, Weighted median, and Inverse variance-weighted (multiplicative random effects), were employed to generate overall estimates of the effect. In order to test whether the associations were likely to be causal, we used inverse variance weighted (IVW) two-sample MR as our primary analysis to estimate the effect of the exposure factors on CRC [23]. The IVW approach utilized a meta-analysis framework to combine Wald estimates for each SNP to derive an overall estimate of the effect, assuming the absence of horizontal pleiotropy, with the IVW results being unbiased. However, if heterogeneity exists, random-effects IVW models are employed. The Maximum likelihood method, similar to IVW, assumes the absence of heterogeneity and horizontal pleiotropy. In the presence of these assumptions, the results will be unbiased, with smaller standard errors compared to IVW. To assess the presence of pleiotropy, the MR-Egger regression approach is utilized, based on the assumption of instrument strength independent of direct effect (InSIDE), allowing for the assessment of the intercept term. A zero-intercept term indicates the absence of horizontal pleiotropy, making the result of the MR-Egger regression consistent with IVW. The weighted median method permits accurate estimation of causal association even when up to 50% of instrumental variables are invalid. When the InSIDE hypothesis is violated, the weighted model estimate has greater power to detect a causal effect, less bias, and lower type I error rates than MR-Egger regression.

The strength of instrumental variables (IVs) is evaluated by computing the F-statistic using the formula F = R2×(N-1-K)/[(1 - R2)×K], where R2 represents the proportion of variance in the exposure explained by the genetic variants, N represents the sample size, and K represents the number of instruments. A corresponding F-statistic greater than 10 is considered indicative of the absence of significant weak instrumental bias [32]. Furthermore, MR-PRESSO corrects for pleiotropy and outlier effects, while the MR Steiger test evaluates the directionality of the causal effect [33]. Heterogeneity and pleiotropy tests assess the homogeneity and pleiotropic effects of instrumental variables, respectively [34]. Finally, leave-one-out analysis tests the robustness of the causal estimates obtained from the MR analysis by iteratively removing each instrumental variable to identify potential heterogeneous SNPs. Together, these methods help to improve the validity and reliability of causal inference in our MR analysis.

### IVW multivariable mendelian randomization

IVW multivariable MR was utilized to assess the causal impact of different liver traits, encompassing liver iron content, percent liver fat, alanine transaminase (ALT) levels, and liver volume, on the risk of colorectal cancer. In this approach, instruments were chosen for each exposure variable. Subsequently, all exposures corresponding to those genetic variants were collectively regressed against the outcome variable, with weighting based on the inverse variance of the outcome. The threshold for instrument inclusion, denoted as pval_threshold, was set at 5.0 × 10^− 8^. Moreover, decisions were made about whether to estimate intercepts or use instrument-specific estimates for each exposure variable. This comprehensive analysis seeks to unravel the potential causal relationships between liver traits and CRC risk while considering the complexities of confounding and bias.

## Results

### Causal effect of liver traits on CRC

Summary statistics of instrumental SNPs as genetic instrumental variables (IVs) for several kinds of liver traits (liver iron content, percent liver fat, alanine transaminase levels and liver volume) were presented in Table S2. These SNPs were not associated with CRC and have no linkage disequilibrium (LD) associations. Each line of the table included 9items related to the SNP, such as the SNPs, effect allele, other allele, beta coefficients and standard error of the SNP on the risk of the corresponding liver traits and CRC.

**Fig. 2:**
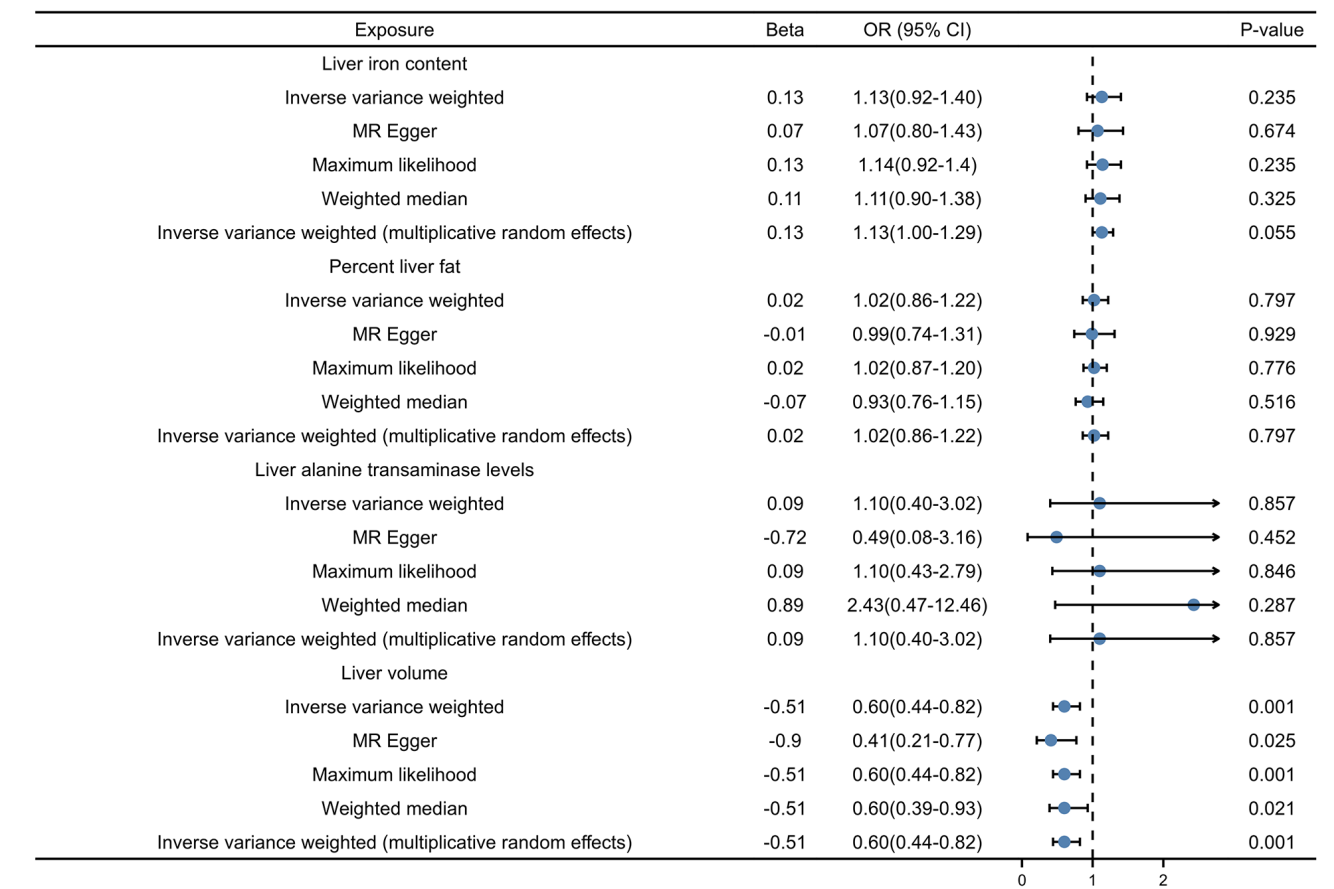
Associations between genetically predicted liver and CRC. CI indicates confidence interval; OR, odds ratio. MR-Egger is able to detect some violations of the standard assumptions of the instrumental variables and provide an estimate of the effect that is not subject to these violations. The weighted median analysis is used to combine data from multiple genetic variants into a single causal estimate.( Liver iron content in mg/g, percent liver fat in %, liver volume in L, Liver enzyme levels (alanine transaminase) in IU/L)

As shown in Fig. 2, our analysis suggested a causal effect between liver volume and a reduced risk of colorectal cancer (the five MR methods demonstrated consistent effect directions and statistical significance with a a Benjamini-Hochberg method-corrected P value < 0.013 and did not provide evidence for causal effects of liver iron content, percent liver fat, or liver alanine transaminase levels. Large liver volumes may represent a decreased risk of CRC (Beta − 0.51; OR 0.60; 95% CI, 0.44–0.82; *P* = 0.0010). The risk of CRC lacked evidence of association with liver iron content, percent liver fat, and liver alanine transaminase levels. Liver iron content (Beta 0.16; OR 1.2; 95% CI, 0.97–1.4; *P* = 0.10), percent liver fat (Beta 0.020; OR 1.0; 95% CI, 0.82–1.2; *P* = 0.79), liver alanine transalanine transase levels (Beta − 0.060; OR 0.94; 95% CI, 0.32–2.8; *P* = 0.91) are provided in Supplemental Fig. 1.

For the causal analysis of liver volume and colorectal cancer risk, the MR-PRESSO method did not identify any outliers (Beta − 0.51; *P* = 0.011; Global Test *P* = 0.47). The MR Steiger test confirmed that the causal direction of the analysis results was correct (*P* = 6.0 × 10^− 101^). And no horizontal pleiotropy was detected according to MR-Egger intercepts, and no evident heterogeneity was identified; detailed information is provided in Table 1. In conclusion, our findings provide tentative evidence for a causal role of liver volume in CRC, although further research is needed to confirm these results.

**Table 1:** Heterogeneity and pleiotropy of individual single nucleotide polymorphisms for Mendelian randomization

Exposure	Heterogeneity		Pleiotropy	
	Cochran’s Q statistic (IVW)	*P* value	MR-Egger intercept	*P* value
Liver iron content	3.0	0.70	0.0021	0.93
Percent liver fat	11	0.30	0.0060	0.75
Liver alanine transaminase levels	2.7 × 10^2^	0.0050	-0.0014	0.88
Liver volume	9.5	0.40	0.032	0.21

The leave-one-out analysis was performed in order to assess the influence of individual SNPs on the estimates. The results demonstrate that the overall values obtained by the leave-one-out analysis were consistent with estimates obtained when all available SNPs were included in the analysis. Further information on the leave-one-out validation results can be found in Supplemental Fig. 2.

The findings derived from the application of IVW multivariable MR exhibited coherence with the outcomes obtained through the Two-sample MR. SNPs employed in IVW multivariable MR and their beta, standard error, and P value for exposure and outcome can be found in Additional File 1. Notably, the analysis revealed a significant and consistent trend for liver volume’s association with colorectal cancer risk. Specifically, liver volume demonstrated a noteworthy protective effect against colorectal cancer onset, as indicated by the estimated Beta coefficient of -0.45 (SE = 0.16, P = 0.0050). A graphical representation of the results is available in Supplemental Fig. 3, showcasing scatter plots generated from the IVW multivariable MR analysis. This alignment between the IVW multivariable MR and the Two-sample MR reinforces the observed connection between liver volume and colorectal cancer risk, further validating the significance of this relationship.

## Discussion

This study provided genetic evidence for a causal relationship between liver volume and CRC risk and found a possible causal role for liver volume in reducing CRC risk. However, there was no evidence for a causal role of liver iron content, percent liver fat, or liver alanine transaminase levels in CRC risk.

The characteristics of the liver are closely related to the onset and progression of many diseases. In diabetic ketoacidosis (DKA) disease, it has been shown that alanine aminotransferase (ALT) has a greater correlation with the disease in patients aged 65 years and older and in obese patients [35]. Other studies have shown a strong causal relationship between higher levels of liver fat and the risk of type 2 diabetes, which is consistent with recent MR studies showing a causal relationship between non-alcoholic fatty liver disease or its markers (ALT and AST) and a higher risk of type 2 diabetes [30].

However, the iron content of the liver does influence the course of the disease. High iron levels are an independent inducer or cofactor of hepatocellular carcinoma (HCC). And there is a correlation between hepatic iron level and hepatocellular carcinoma among patients with end-stage liver disease, while the strongest correlation between hepatic iron level and hepatocellular carcinoma is found among patients with biliary cirrhosis and hepatitis C [36]. Patients with cancer have a history of chemotherapy, which puts them at increased risk of liver toxicity and pancytopenia, leading to elevated liver fat and iron. Moreover,liver volume is another factor that influences disease progression. Despite limitations in younger patients, liver volume remains a traditional imaging biomarker for polycystic liver [37]. In patients with diagnosis of hilar or distal bile duct cancer, liver volume is a useful tool to assess the efficacy of biliary stenting [38]. After gastrectomy in patients with gastric adenocarcinoma, the second and third segments of the liver show significant atrophy compared with the rest of the liver and the whole liver, and the volume reduction is poorly correlated with time [39].

The liver, the most common site of metastasis in CRC, has seen an increase in the number of patients with resectable colorectal liver metastases (CRLM) over the past two decades. However, the reasons for the increase in numbers are due to advances in intensive systemic chemotherapy regimens associated with targeted therapies or immunotherapy, on the one hand, and technical improvements that increase the size of future liver remnant (FLR) and facilitate parenchyma-sparing surgery, on the other [40].

Our results showed that liver volume has a protective effect against CRC. Liver volume, which reflects the number of liver parenchymal cells and is a quantitative measure of liver reserve function from a morphological point of view, is an indicator for assessing disease severity and predicting outcome [41]. The liver consists of approximately 80% hepatocytes and 20% non-hepatocytes [42], and there is a direct relationship between liver volume and cell content. As the most regenerative organ in the body, the liver’s powerful regenerative capacity comes at the expense of the liver’s reserve function [43]. When the liver is injured, there is massive necrosis, shrinkage, and structural collapse of the liver tissue, leading to a reduction in volume and dysfunction of the hepatic reserve [41].

Hepatocytes are the major parenchymal cells responsible for metabolic functions and most circulating plasma proteins such as albumin, transporters, protease inhibitors, coagulation factors and modulators of immune complexes and inflammation are expressed by hepatocytes [44]. They metabolise amino acids, metals and endogenous compounds such as haem and bilirubin, and control the homeostasis of molecules such as glucose/glycogen, triglycerides, cholesterol, bile acids and vitamins A and D [45]. Amino acids are not only building blocks of proteins, they are also intermediate metabolites that power a variety of biosynthetic pathways [46]. Glutamine is an essential amino acid that is used by cancer cells for biosynthetic, bioenergetic and antioxidant purposes [47]. For the bioenergetic and biosynthetic needs of cells, glutamate serves as an important carbon source. When proliferating cells use glutamine-derived carbon to enter the TCA cycle, most of the citrate produced is exported to the cytosol where it is converted to acetyl-CoA, a precursor for the biosynthesis of fatty acids and cholesterol. Oxaloacetate from the TCA cycle is also used to synthesise aspartate and asparagine [48].

In addition to glutamine metabolism, serine and glycine metabolism are also important mediators in cancer cell development [49]. Glycine is a precursor of serine and is involved in a wide range of metabolic processes in both humans and animals as a component of glutathione and as a substrate in the synthesis of purines and proteins [50]. The consumption of glycine and the expression of the mitochondrial glycine biosynthesis pathway are strongly correlated with the rate of proliferation of cancer cells in general and are a source of amino acids required for cancer growth [51, 52]. The primary route for the disposal of glycine is catalysed by the glycine cleavage system, which is located in the inner membrane of the mitochondria in the liver [53]. In contrast, the glycine decarboxylation system of cancer cells can break down glycine in the presence of the glycine decarboxylase complex to form ammonia, carbon dioxide, and methylenetetrahydrofolate [54]. Serine catabolism is initiated by the interconversion of serine to glycine, producing a large number of one-carbon units that activate one-carbon metabolism, forming an intracellular double-loop pathway linking the methionine and folate cycles via methionine synthase. The glycine cleavage system and choline catabolism are additional pathways to one-carbon metabolism, and the preferred carbon source driving one-carbon metabolism in cancer cells is the uptake and subsequent breakdown of glucose into various biomass precursors, including the synthesis of serine and glycine [55].In macromolecular synthesis, serine can load transfer RNAs (tRNAs) for protein synthesis, serve as a precursor for amino acids such as cysteine and glycine, provide head groups for sphingolipid and phospholipid synthesis, or donate 1 C units for nucleotide synthesis [56].Thus, the volume of the liver is determined by the hepatocytes, which have the role of metabolizing amino acids. Amino acid metabolism is an important mediator of cancer development and may have the same effect on colorectal cancer.

In summary, these findings provide insights into the biological mechanisms underlying the complex relationship between liver characteristics and CRC development, and may inform the development of targeted therapeutic interventions for patients with CRLM. Our study highlights the importance of MR as a powerful epidemiological tool for investigating causal associations between exposures and outcomes. However, there are some limitations to this study that should be considered. First, although we examined the effect of certain circulating liver characteristics on CRC, it was not possible to examine possible non-linear relationships with CRC. Another limitation is that the associations between liver traits and CRC were derived from a European cohort, making it difficult to extrapolate to other populations. An increase in the diversity of the populations studied is required for even modest differences between populations in the contribution of shared variation to complex traits [57]. Therefore, the results we present can only be extended to European populations. What’s more, in contrasting the covariate adjustments between the cohorts, it’s evident that the “Liver iron content” cohort incorporates specific imaging-related covariates, such as the imaging center, scan date, and scan time, which are absent in other cohorts, because it is a radiological feature. Additionally, while the “Liver alanine transaminase levels” cohort adjusts for a notable 40 genetic principal components, the “Colorectal cancer” cohort includes adjustments for just 10 genetic principal components and “Liver iron content” doesn’t consider PCs as a covariate. Such differences in covariate adjustments can have implications. For instance, the variance in the number of genetic principal components used between cohorts suggests that there might be different degrees of control for potential genetic confounding. These disparities underscore the importance of being cautious when interpreting results, especially in cross-cohort comparisons. Additionally, the demographic disparities within the dataset present a critical issue for consideration. It is evident that three of the datasets (Liver iron content, Percent liver fat, and Liver volume) predominantly encompass cohorts from the UK Biobank, consisting of individuals of European ancestry, meticulously ascertained based on self-identified “White British” ancestry. In contrast, the cohort for Liver alanine transaminase levels consists of a discovery sample drawn from individuals of European descent from the UK Biobank, while the replication sample encompasses individuals of European lineage from both the Netherlands and the United States. Furthermore, the cohorts for Colorectal cancer are exclusively composed of Finnish individuals. Notably, while the exposure groups exhibit uniformity in composition and origin, predominantly from the UK Biobank (excluding the replication sample), the outcome population originates from Finland. Even though both populations are of European descent, the potential biases introduced by their distinct backgrounds in Mendelian randomization cannot be dismissed. For future endeavors, it would be prudent to employ datasets with more congruent demographic backgrounds to validate these findings, ensuring their robustness.

Larger and more comprehensive MR studies are warranted to better understand the intrinsic relationship between liver traits and CRC. More research is needed to clarify the association between hepatic characteristics and the risk of CRC and to explore the underlying mechanisms.

### Electronic supplementary material

Below is the link to the electronic supplementary material.

Supplementary Material 1

Supplementary Material 2

## Data Availability

The datasets generated and/or analyzed during the current study are available in the [GWAS summary data] repository, [Liver iron content: https://gwas.mrcieu.ac.uk/datasets/ebi-a-GCST90016674/, percent liver fat: https://gwas.mrcieu.ac.uk/datasets/ebi-a-GCST90016673/, alanine transaminase levels: https://gwas.mrcieu.ac.uk/datasets/ebi-a-GCST004940/, liver volume: https://gwas.mrcieu.ac.uk/datasets/ebi-a-GCST90016666/, Colorectal Cancer: https://gwas.mrcieu.ac.uk/datasets/finn-b-C3_COLORECTAL_EXALLC/]
