# Supplementary material for: Causal associations between liver traits and Colorectal cancer: a Mendelian randomization study
Source: BMC Med Genomics. 2023 Dec 6;16:316. doi: 10.1186/s12920-023-01755-w (PMC10699049; doi:10.1186/s12920-023-01755-w)
Supplement: Supplementary file 1 — Supplementary Material 1 [file 12920_2023_1755_MOESM1_ESM.docx]

**Characterization of datasets employed in this study**

Part 1 Data source and sample size of 5 datasets

| Trait | GWAS ID | Link | Sample Size |
| --- | --- | --- | --- |
| Liver iron content | ebi-a-GCST90016674 | https://gwas.mrcieu.ac.uk/datasets/ebi-a-GCST90016674/ | 32,858 |
| Percent liver fat | ebi-a-GCST90016673 | https://gwas.mrcieu.ac.uk/datasets/ebi-a-GCST90016673/ | 32,858 |
| Liver volume | ebi-a-GCST90016666 | https://gwas.mrcieu.ac.uk/datasets/ebi-a-GCST90016666/ | 32,860 |
| Liver enzyme levels (alanine transaminase) | ebi-a-GCST90013405 | https://gwas.mrcieu.ac.uk/datasets/ebi-a-GCST90013405/ | 437,267 |
| Colorectal cancer | finn-b-C3_COLORECTAL | https://gwas.mrcieu.ac.uk/datasets/finn-b-C3_COLORECTAL/ | 218,792 |

Part 2 Details and characterization of 5 datasets

Three datasets, including ebi-a-GCST90016674, ebi-a-GCST90016673, and ebi-a-GCST90016666, involve substantial cohorts of 32,858, 32,858 and 32,860 individuals of European ancestry from the United Kingdom respectively, meticulously selected based on self-reported "White British" ancestry and verified through principal components analysis. Rigorous quality control procedures were applied, eliminating participants with sex chromosome anomalies, genetic inconsistencies, and outliers in terms of heterozygosity and genotype call rate. The genotyping technology employed was a genome-wide genotyping array, specifically Affymetrix, with approximately 9,390,170 SNPs that passed the quality control threshold (MAF ≥ 0.01 and INFO ≥ 0.9) used for the analysis. This investigation delves into the genetic architecture of Liver iron content, Percent liver fat and Liver volume, drawing on advanced techniques including deep learning algorithms. The study's robust analysis employed BOLT-LMM version 2.3.2, accounting for various covariates such as age, sex, imaging factors, and genotyping batches. The genetic relatedness matrix was incorporated as a random-effect, ensuring a comprehensive examination of the trait's genetic underpinnings. The research, led by Liu Y and collaborating authors, was published in the journal eLife on June 15, 2021[1]. The population background and detailed information are depicted in Table 1.

|  | UK biobank cohort (at time of baseline visit) | Imaging cohort (at time of imaging visit) | GWAS cohort (White British Ancestry and passing QC) | |
| --- | --- | --- | --- | --- |
|  |  |  | Liver volume (DIXON) | Liver fat and iron |
| Number of participants | 502,520 | 38,881* | 32,860 | 32,858 |
| % Female | 54.4 | 51.8 | 51.5 | 51.5 |
| Age | 56.5 (8.1) | 63.7 (7.56) | 63.9 (7.52) | 63.9 (7.52) |
| BMI (kg/m2) | 27.4 (4.8) | 26.5 (4.39) | 26.5 (4.37) | 26.5 (4.36) |
| Height (cm) | 168 (9.28) | 169 (9.3) | 169 (9.26) | 169 (9.26) |
| % White British Ancestry | 81.5 | 81.5 | 100 | 100 |

**Table 1.** Age, BMI, and height rows give mean and SD for each population. (Number of imaging participants gives the number with at least one abdominal IDP successfully extracted.)

The dataset ebi-a-GCST90013405 focused on the investigation of genetic factors associated with liver enzyme levels, specifically serum alanine aminotransferase (ALT) measurement. The study involves a substantial discovery sample of 437,267 individuals of European ancestry, aged between 40 and 69 years, recruited from various centers across the United Kingdom. The participants were selected based on stringent quality measures, including exclusions for sex discordance, high missingness, and heterozygosity. The dataset leverages data from the UK Biobank and employs state-of-the-art genotyping technology, utilizing a genome-wide genotyping array to analyze a large number of single nucleotide polymorphisms (SNPs). To ensure the robustness of their findings, the study also involves a replication stage that encompasses 315,572 individuals from independent studies in Europe and the United States. Additionally, genetic risk scores (GRS) were used to further validate the association of the identified SNPs with serum levels of ALT, as well as other liver enzymes (ALP and GGT), in separate samples from the Airwave health monitoring study and the Northern Finland Birth Cohort 1966. The dataset is characterized by its comprehensive methodology, involving both discovery and replication stages, stringent participant selection criteria, thorough quality control measures, and the utilization of advanced genetic analysis techniques. The study's findings contribute to the understanding of genetic factors influencing liver enzyme levels, potentially shedding light on the molecular mechanisms underlying liver health and function. This Genome-wide Association Study, detailed in the publication by Pazoki et al. in the journal "Nature Communications"[2].

The dataset finn-b-C3_COLORECTAL encompasses primary and metastatic malignant neoplasms affecting the colon or rectum, including carcinoma, lymphoma, and sarcoma. The dataset includes phenotype data from 4,736 individuals, with no specific sex-based rules or conditions applied. It involves various endpoints such as C3_COLON, C3_RECTOSIGMOID_JUNCTION, and C3_RECTUM from the FinnGen project, ensuring comprehensive coverage of the condition's manifestations. The dataset incorporates data from 9,132 cases and 8,801 controls, where controls are individuals without colorectal cancer. The dataset's utility is underscored by its utilization in the FinnGen project and its exclusion from FinRegistry analyses. The summary statistics showcase a notable prevalence of 2.10% across all individuals, with slight variations between female (1.45%) and male (2.57%) participants. The median age at the first recorded event was approximately 68.50 years. See its full statistics at the FinnGen website (<https://risteys.finregistry.fi/endpoints/C3_COLORECTAL>).

Part 3 Summary of the differences and similarities of 5 datasets

Here's a summary of the differences and similarities in terms of population source, age, and gender for these five datasets:

**ebi-a-GCST90016674, ebi-a-GCST90016673, ebi-a-GCST90016666 datasets:**

Population Source: All datasets comprise individuals of European ancestry from the United Kingdom.

Age: The average age is approximately 63.9 years across all datasets.

Gender: Female representation is around 51.5% in all three datasets.

**ebi-a-GCST90013405 dataset:**

Population Source: Individuals of European ancestry aged 40 to 69 years, recruited from various centers across the United Kingdom.

Age: Age range is 40 to 69 years.

Gender: Gender distribution not explicitly provided.

**finn-b-C3_COLORECTAL dataset:**

Population Source: No specific sex-based rules; includes individuals with primary and metastatic colorectal malignancies.

Age: Median age at first recorded event is approximately 68.5 years.

Gender: Slight gender variations, with prevalence in males (2.57%) higher than in females (1.45%).

In summary, all datasets involve individuals of European ancestry. The age distribution varies, with the ebi-a-GCST90013405 dataset focusing on individuals aged 40 to 69, while the other datasets have an average age of around 63.9 years. Gender distribution is generally balanced across the datasets, except for the finn-b-C3_COLORECTAL dataset, which shows slight differences in prevalence between males and females.

| Cohort | Trait | Covariates |
| --- | --- | --- |
| ebi-a-GCST90016674 | Liver iron content | age at imaging visit, age squared, sex, imaging centre, scan date, scan time, and genotyping batch |
| ebi-a-GCST90016673 | Percent liver fat |  |
| ebi-a-GCST90016666 | Liver volume |  |
| ebi-a-GCST90013405 | Liver alanine transaminase levels | age, sex, 40 genetic principal components and array batch |
| finn-b-C3_COLORECTAL | Colorectal cancer | age, sex, 10 genetic principal components and genotyping batch |

**Supplemental Table 1.** Covariates adjusted for the GWAS cohorts.

**Supplemental Table 2.** Characteristics of selected SNP instrumental variables for liver traits.

| SNP |  | | Liver traits | | | Colorectal cancer | | |
| --- | --- | --- | --- | --- | --- | --- | --- | --- |
|  | effect allele | other allele | beta | se | p-val | beta | se | p-val |
| Liver iron content |  |  |  |  |  |  |  |  |
| rs115380467 | A | G | 0.1632 | 0.0259 | 3.0E-10 | 0.0876 | 0.0905 | 0.33 |
| rs2690093 | C | G | 0.2296 | 0.0128 | 2.4E-72 | 0.0287 | 0.0353 | 0.42 |
| rs2690093 | C | G | 0.2296 | 0.0128 | 2.4E-72 | 0.0960 | 0.0626 | 0.13 |
| rs7577758 | T | C | 0.0606 | 0.0092 | 3.6E-11 | 0.0307 | 0.0297 | 0.30 |
| rs80215559 | C | T | 0.5492 | 0.0145 | 1.0E-200 | 0.0543 | 0.0690 | 0.43 |
| rs855791 | G | A | 0.0901 | 0.0078 | 8.3E-31 | 0.0129 | 0.0279 | 0.64 |
| rs9268652 | G | A | -0.0565 | 0.0089 | 2.0E-10 | 0.0195 | 0.0301 | 0.52 |
| Percent liver fat |  |  |  |  |  |  |  |  |
| rs10787429 | C | T | -0.0604 | 0.0086 | 2.8E-12 | 0.0080 | 0.0287 | 0.78 |
| rs112875651 | A | G | -0.0514 | 0.0079 | 9.3E-11 | -0.0686 | 0.0274 | 0.012 |
| rs188247550 | T | C | 0.3363 | 0.0334 | 6.7E-24 | -0.0229 | 0.0630 | 0.72 |
| rs2642438 | G | A | 0.0545 | 0.0084 | 9.6E-11 | -0.0106 | 0.0296 | 0.75 |
| rs429358 | C | T | -0.1230 | 0.0107 | 1.0E-30 | 0.0151 | 0.0354 | 0.67 |
| rs4665985 | C | A | 0.0495 | 0.0087 | 1.5E-08 | -0.0143 | 0.0315 | 0.65 |
| rs58542926 | T | C | 0.3334 | 0.0145 | 2.5E-116 | -0.0256 | 0.0542 | 0.64 |
| rs7029757 | A | G | -0.0766 | 0.0131 | 4.8E-09 | -0.0510 | 0.0557 | 0.36 |
| rs738409 | G | C | 0.2295 | 0.0093 | 1.5E-133 | 0.0340 | 0.0318 | 0.28 |
| rs7689584 | G | T | -0.0529 | 0.0088 | 1.9E-09 | 0.0416 | 0.0334 | 0.21 |
| Liver alanine transaminase levels |  |  |  |  |  |  |  |  |
| rs10075805 | G | A | -0.0056 | 0.0004 | 3.9E-38 | -0.0200 | 0.0287 | 0.49 |
| rs1030270 | T | C | -0.0065 | 0.0008 | 6.4E-15 | 0.0377 | 0.0402 | 0.35 |
| rs10420309 | G | A | -0.0023 | 0.0004 | 4.1E-09 | -0.0199 | 0.0272 | 0.46 |
| rs10774169 | G | A | -0.0024 | 0.0004 | 2.6E-08 | 0.0661 | 0.0283 | 0.020 |
| rs1077835 | G | A | -0.0035 | 0.0005 | 1.1E-13 | -0.0124 | 0.0306 | 0.69 |
| rs10787429 | C | T | -0.0058 | 0.0004 | 2.8E-39 | 0.0080 | 0.0287 | 0.78 |
| rs10833712 | G | T | 0.0028 | 0.0004 | 3.7E-13 | -0.0164 | 0.0270 | 0.54 |
| rs10841520 | T | C | -0.0032 | 0.0005 | 4.0E-11 | -0.0335 | 0.0311 | 0.28 |
| rs10887777 | C | T | 0.0030 | 0.0004 | 2.1E-11 | 0.0337 | 0.0318 | 0.29 |
| rs10900229 | T | C | -0.0026 | 0.0004 | 4.9E-09 | 0.0087 | 0.0287 | 0.76 |
| rs10913481 | G | A | 0.0025 | 0.0005 | 1.3E-08 | -0.0184 | 0.0322 | 0.57 |
| rs10971930 | C | T | 0.0038 | 0.0006 | 1.1E-10 | 0.0584 | 0.0395 | 0.14 |
| rs11002308 | T | A | 0.0048 | 0.0004 | 1.2E-31 | -0.0136 | 0.0298 | 0.65 |
| rs1107364 | A | G | 0.0024 | 0.0004 | 2.9E-09 | -0.0084 | 0.0267 | 0.75 |
| rs111655426 | A | G | -0.0204 | 0.0016 | 2.1E-37 | -0.0438 | 0.0590 | 0.46 |
| rs11167753 | C | T | 0.0023 | 0.0004 | 3.8E-08 | -0.0081 | 0.0296 | 0.78 |
| rs112574791 | A | G | -0.0408 | 0.0017 | 3.6E-124 | -0.0614 | 0.1239 | 0.62 |
| rs114165349 | C | G | 0.0170 | 0.0013 | 2.2E-39 | -0.0277 | 0.0717 | 0.70 |
| rs11429307 | GT | G | 0.0051 | 0.0005 | 6.6E-24 | 0.0143 | 0.0394 | 0.72 |
| rs11433460 | GT | G | -0.0031 | 0.0005 | 2.1E-08 | -0.0010 | 0.0406 | 0.98 |
| rs11543269 | T | C | -0.0068 | 0.0006 | 1.0E-30 | 0.0617 | 0.0409 | 0.13 |
| rs11582968 | A | T | 0.0060 | 0.0007 | 1.6E-15 | 0.0459 | 0.0674 | 0.50 |
| rs11601507 | A | C | 0.0081 | 0.0008 | 2.1E-27 | -0.0280 | 0.0498 | 0.57 |
| rs11621792 | T | C | 0.0036 | 0.0004 | 4.0E-19 | -0.0233 | 0.0276 | 0.40 |
| rs11657440 | C | T | 0.0046 | 0.0004 | 1.1E-32 | 0.0325 | 0.0269 | 0.23 |
| rs11727676 | C | T | 0.0049 | 0.0007 | 1.0E-13 | 0.1412 | 0.0474 | 0.0029 |
| rs117630094 | C | T | 0.0124 | 0.0021 | 9.8E-09 | 0.0991 | 0.0886 | 0.26 |
| rs117643180 | A | C | 0.0107 | 0.0012 | 4.2E-18 | -0.1919 | 0.0881 | 0.029 |
| rs11770163 | C | G | 0.0028 | 0.0004 | 1.6E-12 | -0.0599 | 0.0278 | 0.031 |
| rs11837319 | G | C | -0.0029 | 0.0004 | 1.5E-11 | -0.0181 | 0.0333 | 0.59 |
| rs11875971 | C | A | -0.0025 | 0.0005 | 2.4E-08 | -0.0371 | 0.0279 | 0.18 |
| rs11938781 | C | T | 0.0030 | 0.0005 | 5.8E-09 | -0.0002 | 0.0430 | 1.0 |
| rs11986065 | A | G | 0.0035 | 0.0005 | 1.4E-14 | 0.0539 | 0.0297 | 0.070 |
| rs12202204 | G | A | 0.0022 | 0.0004 | 4.6E-08 | 0.0037 | 0.0302 | 0.90 |
| rs12320328 | G | A | -0.0042 | 0.0007 | 6.2E-10 | -0.0360 | 0.0377 | 0.34 |
| rs12405515 | T | G | -0.0022 | 0.0004 | 1.9E-08 | 0.0102 | 0.0269 | 0.70 |
| rs12414592 | C | A | -0.0044 | 0.0005 | 3.3E-19 | 0.0500 | 0.0359 | 0.16 |
| rs12432645 | T | G | 0.0030 | 0.0004 | 4.8E-12 | -0.0030 | 0.0303 | 0.92 |
| rs12442886 | C | T | 0.0026 | 0.0005 | 1.1E-08 | -0.0306 | 0.0314 | 0.33 |
| rs12484801 | T | C | 0.0197 | 0.0005 | 1.0E-200 | 0.0271 | 0.0339 | 0.42 |
| rs12500824 | G | A | -0.0038 | 0.0004 | 3.1E-21 | -0.0462 | 0.0280 | 0.10 |
| rs12516167 | C | T | -0.0056 | 0.0010 | 3.5E-08 | -0.0693 | 0.0498 | 0.16 |
| rs12609548 | A | T | -0.0024 | 0.0004 | 4.0E-10 | 0.0197 | 0.0267 | 0.46 |
| rs12792832 | G | C | 0.0045 | 0.0008 | 1.7E-08 | -0.0588 | 0.0752 | 0.43 |
| rs12894146 | A | C | 0.0034 | 0.0004 | 9.9E-19 | -0.0179 | 0.0267 | 0.50 |
| rs12904 | A | G | -0.0053 | 0.0004 | 5.2E-43 | -0.0246 | 0.0266 | 0.35 |
| rs12946564 | A | G | -0.0025 | 0.0004 | 1.3E-08 | -0.0182 | 0.0308 | 0.55 |
| rs12979186 | T | C | 0.0025 | 0.0004 | 1.4E-11 | 0.0284 | 0.0271 | 0.30 |
| rs13026251 | T | C | 0.0026 | 0.0004 | 9.5E-11 | 0.0052 | 0.0279 | 0.85 |
| rs13062221 | C | T | -0.0022 | 0.0004 | 2.8E-08 | -0.0596 | 0.0275 | 0.030 |
| rs13083375 | T | G | -0.0069 | 0.0006 | 7.1E-32 | -0.0025 | 0.0352 | 0.94 |
| rs13108218 | G | A | -0.0031 | 0.0004 | 9.2E-15 | -0.0528 | 0.0286 | 0.065 |
| rs13234407 | A | G | -0.0023 | 0.0004 | 4.2E-09 | 0.0350 | 0.0266 | 0.19 |
| rs132642 | T | A | 0.0085 | 0.0005 | 5.5E-60 | -0.0179 | 0.0398 | 0.65 |
| rs13273592 | C | T | 0.0030 | 0.0004 | 9.4E-13 | 0.0701 | 0.0298 | 0.019 |
| rs13275089 | C | T | -0.0033 | 0.0004 | 1.6E-17 | 0.0058 | 0.0285 | 0.84 |
| rs133015 | G | C | -0.0032 | 0.0004 | 2.0E-16 | -0.0160 | 0.0270 | 0.55 |
| rs1332329 | C | A | -0.0023 | 0.0004 | 2.1E-08 | -0.0297 | 0.0270 | 0.27 |
| rs13389219 | T | C | -0.0045 | 0.0004 | 1.7E-30 | -0.0306 | 0.0280 | 0.27 |
| rs13395911 | T | A | 0.0061 | 0.0004 | 5.1E-53 | -0.0586 | 0.0268 | 0.029 |
| rs140517069 | T | G | 0.0078 | 0.0014 | 4.7E-08 | -0.1532 | 0.0611 | 0.012 |
| rs141117801 | T | C | -0.0062 | 0.0006 | 3.7E-22 | 0.0226 | 0.0500 | 0.65 |
| rs143001834 | A | G | 0.0054 | 0.0008 | 2.1E-11 | -0.0213 | 0.0820 | 0.80 |
| rs145158805 | ATGGACATCTAG | A | 0.0032 | 0.0005 | 4.9E-09 | -0.0325 | 0.0365 | 0.37 |
| rs1461729 | G | A | -0.0090 | 0.0006 | 2.0E-44 | 0.0727 | 0.0399 | 0.069 |
| rs1487445 | T | C | -0.0022 | 0.0004 | 2.6E-09 | 0.0359 | 0.0266 | 0.18 |
| rs1497406 | G | A | 0.0071 | 0.0004 | 4.7E-72 | -0.0246 | 0.0285 | 0.39 |
| rs151068477 | T | G | 0.0139 | 0.0022 | 2.3E-10 | 0.1839 | 0.1101 | 0.095 |
| rs1594320 | C | G | -0.0042 | 0.0004 | 1.1E-22 | 0.0100 | 0.0307 | 0.75 |
| rs17138478 | A | C | -0.0057 | 0.0006 | 4.9E-23 | 0.0272 | 0.0368 | 0.46 |
| rs17145738 | T | C | 0.0061 | 0.0006 | 3.9E-25 | 0.0594 | 0.0397 | 0.13 |
| rs17195603 | T | C | -0.0045 | 0.0007 | 7.3E-12 | 0.1079 | 0.0493 | 0.029 |
| rs17532490 | A | G | -0.0041 | 0.0007 | 2.0E-09 | -0.0438 | 0.0613 | 0.48 |
| rs17580 | A | T | 0.0057 | 0.0009 | 2.6E-10 | 0.0827 | 0.1396 | 0.55 |
| rs17710008 | A | G | 0.0031 | 0.0005 | 1.1E-09 | -0.0213 | 0.0398 | 0.59 |
| rs1778793 | G | C | -0.0032 | 0.0004 | 2.8E-16 | 0.0147 | 0.0267 | 0.58 |
| rs1801689 | C | A | 0.0145 | 0.0011 | 1.7E-39 | 0.0094 | 0.1358 | 0.95 |
| rs181450148 | A | G | 0.0146 | 0.0016 | 1.7E-19 | -0.0355 | 0.1325 | 0.79 |
| rs188247550 | T | C | 0.0190 | 0.0018 | 8.7E-27 | -0.0229 | 0.0630 | 0.72 |
| rs1882862 | T | C | 0.0042 | 0.0006 | 6.0E-12 | 0.0324 | 0.0382 | 0.40 |
| rs1890426 | C | T | 0.0032 | 0.0004 | 5.8E-16 | 0.0085 | 0.0269 | 0.75 |
| rs1892535 | A | G | 0.0036 | 0.0005 | 7.0E-13 | 0.0016 | 0.0283 | 0.96 |
| rs1929848 | T | G | -0.0035 | 0.0006 | 7.6E-11 | 0.0589 | 0.0464 | 0.20 |
| rs2000469 | G | A | -0.0025 | 0.0004 | 2.1E-10 | -0.0239 | 0.0286 | 0.40 |
| rs2001613 | T | C | 0.0023 | 0.0004 | 5.6E-10 | 0.0116 | 0.0270 | 0.6 |
| rs2114271 | G | A | -0.0033 | 0.0005 | 6.8E-11 | 0.0418 | 0.0348 | 0.23 |
| rs2127015 | C | T | 0.0038 | 0.0004 | 2.8E-22 | -0.0605 | 0.0268 | 0.024 |
| rs217184 | C | T | -0.0047 | 0.0005 | 1.1E-22 | -0.0352 | 0.0325 | 0.28 |
| rs2229742 | C | G | 0.0045 | 0.0006 | 2.9E-12 | 0.0294 | 0.0421 | 0.48 |
| rs2241160 | G | A | -0.0022 | 0.0004 | 7.6E-09 | -0.0197 | 0.0271 | 0.47 |
| rs2250127 | A | G | -0.0029 | 0.0005 | 1.1E-10 | 0.0573 | 0.0364 | 0.12 |
| rs235314 | T | C | -0.0032 | 0.0004 | 5.4E-17 | -0.0141 | 0.0268 | 0.60 |
| rs2356864 | A | G | 0.0027 | 0.0004 | 7.7E-12 | -0.0732 | 0.0285 | 0.010 |
| rs236651 | A | G | 0.0026 | 0.0004 | 8.5E-11 | -0.0081 | 0.0267 | 0.76 |
| rs2544645 | C | G | -0.0030 | 0.0004 | 7.7E-12 | -0.0381 | 0.0317 | 0.23 |
| rs2642438 | G | A | 0.0068 | 0.0004 | 6.2E-57 | -0.0106 | 0.0296 | 0.72 |
| rs2664139 | C | T | 0.0027 | 0.0004 | 2.5E-10 | -0.0108 | 0.0315 | 0.73 |
| rs2686378 | A | G | 0.0031 | 0.0004 | 2.6E-14 | -0.0063 | 0.0269 | 0.82 |
| rs2710804 | C | T | -0.0028 | 0.0004 | 1.2E-11 | -0.0166 | 0.0278 | 0.55 |
| rs2738759 | G | A | 0.0042 | 0.0007 | 1.1E-09 | -0.0866 | 0.0542 | 0.11 |
| rs2812208 | C | G | -0.0103 | 0.0014 | 1.9E-14 | -0.0394 | 0.0906 | 0.66 |
| rs2836755 | T | C | 0.0025 | 0.0004 | 1.5E-10 | -0.0148 | 0.0267 | 0.58 |
| rs28431971 | A | G | -0.0033 | 0.0004 | 9.6E-13 | 0.0398 | 0.0335 | 0.24 |
| rs28471687 | G | A | 0.0050 | 0.0007 | 1.4E-12 | 0.0295 | 0.0711 | 0.68 |
| rs2862954 | C | T | -0.0121 | 0.0004 | 1.0E-200 | -0.0116 | 0.0273 | 0.67 |
| rs2862996 | T | G | -0.0025 | 0.0004 | 3.1E-09 | 0.0276 | 0.0289 | 0.34 |
| rs28650012 | C | G | -0.0049 | 0.0004 | 3.4E-30 | -0.0027 | 0.0297 | 0.93 |
| rs2874282 | A | G | -0.0028 | 0.0004 | 1.0E-12 | 0.0011 | 0.0268 | 0.97 |
| rs288139 | A | G | -0.0031 | 0.0005 | 9.9E-11 | -0.0353 | 0.0302 | 0.24 |
| rs28929474 | T | C | 0.0231 | 0.0014 | 3.8E-63 | -0.1598 | 0.0955 | 0.094 |
| rs2943654 | T | C | 0.0052 | 0.0004 | 1.1E-38 | -0.0297 | 0.0276 | 0.28 |
| rs2954021 | G | A | -0.0081 | 0.0004 | 4.5E-98 | -0.0129 | 0.0266 | 0.63 |
| rs2963431 | T | C | 0.0025 | 0.0005 | 4.9E-08 | -0.0242 | 0.0363 | 0.50 |
| rs2980682 | T | A | 0.0033 | 0.0004 | 1.8E-15 | 0.0037 | 0.0280 | 0.89 |
| rs3123129 | C | T | -0.0026 | 0.0004 | 4.8E-11 | 0.0051 | 0.0275 | 0.85 |
| rs3184504 | C | T | -0.0045 | 0.0004 | 5.8E-31 | 0.0420 | 0.0270 | 0.12 |
| rs339969 | A | C | 0.0038 | 0.0004 | 1.2E-22 | 0.0483 | 0.0296 | 0.10 |
| rs34696920 | A | C | -0.0030 | 0.0005 | 8.2E-09 | 0.0157 | 0.0355 | 0.66 |
| rs35401514 | TC | T | 0.0046 | 0.0004 | 8.5E-28 | -0.0481 | 0.0297 | 0.10 |
| rs35742417 | A | C | -0.0033 | 0.0005 | 8.5E-12 | 0.0338 | 0.0323 | 0.30 |
| rs35747256 | CT | C | -0.0024 | 0.0004 | 1.2E-09 | -0.0342 | 0.0274 | 0.21 |
| rs3731714 | T | C | -0.0027 | 0.0004 | 7.0E-11 | 0.0162 | 0.0288 | 0.57 |
| rs3794701 | G | A | 0.0024 | 0.0004 | 7.0E-10 | 0.0050 | 0.0269 | 0.85 |
| rs3795445 | C | A | 0.0021 | 0.0004 | 2.6E-08 | 0.0117 | 0.0270 | 0.66 |
| rs3824488 | T | C | -0.0037 | 0.0007 | 1.6E-08 | -0.0094 | 0.0438 | 0.83 |
| rs3837067 | AT | A | 0.0030 | 0.0004 | 4.6E-12 | 0.0146 | 0.0299 | 0.63 |
| rs3847263 | C | T | 0.0022 | 0.0004 | 1.9E-08 | -0.0619 | 0.0268 | 0.02 |
| rs4074793 | G | A | 0.0055 | 0.0007 | 1.9E-14 | 0.0296 | 0.0516 | 0.57 |
| rs4075185 | C | G | 0.0025 | 0.0004 | 2.8E-09 | 0.0023 | 0.0301 | 0.94 |
| rs4077189 | A | G | -0.0042 | 0.0007 | 6.6E-10 | 0.0331 | 0.0481 | 0.49 |
| rs4084164 | G | A | -0.0033 | 0.0004 | 1.1E-16 | -0.0304 | 0.0275 | 0.27 |
| rs429358 | C | T | -0.0085 | 0.0005 | 1.1E-56 | 0.0151 | 0.0354 | 0.67 |
| rs4323719 | T | C | 0.0028 | 0.0004 | 1.1E-12 | 0.0051 | 0.0270 | 0.85 |
| rs438568 | G | A | -0.0035 | 0.0004 | 1.1E-18 | -0.0170 | 0.0282 | 0.55 |
| rs445 | T | C | 0.0069 | 0.0007 | 1.5E-26 | 0.1010 | 0.0570 | 0.076 |
| rs4503880 | C | T | -0.0080 | 0.0005 | 4.2E-63 | 0.0154 | 0.0318 | 0.63 |
| rs4647222 | C | A | -0.0030 | 0.0004 | 4.0E-15 | -0.0456 | 0.0275 | 0.097 |
| rs4711456 | T | C | 0.0022 | 0.0004 | 3.5E-08 | -0.0664 | 0.0287 | 0.02 |
| rs4711750 | A | T | 0.0032 | 0.0004 | 3.1E-16 | 0.0319 | 0.0269 | 0.24 |
| rs4782568 | G | C | -0.0044 | 0.0004 | 4.6E-30 | 0.0314 | 0.0270 | 0.24 |
| rs4806498 | T | C | 0.0024 | 0.0004 | 1.6E-09 | 0.0372 | 0.0272 | 0.17 |
| rs4835265 | A | C | 0.0097 | 0.0005 | 7.7E-76 | -0.0443 | 0.0355 | 0.21 |
| rs488218 | T | C | -0.0045 | 0.0007 | 8.6E-10 | -0.0859 | 0.0443 | 0.053 |
| rs4925546 | G | A | -0.0034 | 0.0004 | 8.2E-17 | 0.0085 | 0.0276 | 0.76 |
| rs4948099 | A | C | -0.0027 | 0.0005 | 4.4E-10 | -0.0191 | 0.0353 | 0.59 |
| rs4951163 | C | T | 0.0042 | 0.0007 | 2.1E-09 | 0.0504 | 0.0492 | 0.31 |
| rs4976033 | G | A | 0.0028 | 0.0004 | 1.8E-12 | 0.0054 | 0.0271 | 0.84 |
| rs505922 | C | T | 0.0049 | 0.0004 | 1.8E-32 | 0.0295 | 0.0268 | 0.27 |
| rs519790 | G | C | 0.0039 | 0.0004 | 3.4E-22 | -0.0176 | 0.0300 | 0.56 |
| rs55747905 | C | T | -0.0060 | 0.0005 | 4.5E-33 | 0.0225 | 0.0356 | 0.53 |
| rs56094641 | G | A | 0.0035 | 0.0004 | 1.5E-18 | 0.0126 | 0.0270 | 0.64 |
| rs571174 | C | T | 0.0024 | 0.0004 | 8.4E-09 | 0.0212 | 0.0290 | 0.47 |
| rs5752776 | G | A | 0.0042 | 0.0004 | 8.5E-25 | 0.0328 | 0.0305 | 0.28 |
| rs58542926 | T | C | 0.0171 | 0.0007 | 1.8E-119 | -0.0256 | 0.0542 | 0.63 |
| rs599839 | A | G | 0.0025 | 0.0005 | 1.8E-08 | -0.0213 | 0.0322 | 0.51 |
| rs60140950 | C | G | 0.0050 | 0.0005 | 2.7E-20 | 0.0296 | 0.0422 | 0.48 |
| rs6034011 | C | T | 0.0031 | 0.0004 | 4.4E-13 | 0.0393 | 0.0291 | 0.18 |
| rs60856912 | T | G | 0.0045 | 0.0005 | 3.7E-17 | 0.0121 | 0.0325 | 0.71 |
| rs6132835 | G | A | 0.0026 | 0.0004 | 1.8E-11 | -0.0197 | 0.0267 | 0.46 |
| rs61856806 | A | T | 0.0038 | 0.0005 | 3.5E-14 | 0.0090 | 0.0350 | 0.80 |
| rs62505473 | G | C | 0.0022 | 0.0004 | 1.9E-08 | -0.0044 | 0.0272 | 0.87 |
| rs62523081 | A | G | 0.0025 | 0.0004 | 4.9E-10 | -0.0191 | 0.0272 | 0.48 |
| rs625899 | C | T | -0.0032 | 0.0004 | 2.6E-13 | 0.0370 | 0.0293 | 0.21 |
| rs631695 | G | T | 0.0023 | 0.0004 | 2.1E-09 | -0.0023 | 0.0271 | 0.93 |
| rs645040 | T | G | 0.0054 | 0.0005 | 1.4E-31 | 0.0321 | 0.0372 | 0.39 |
| rs6557781 | C | T | -0.0042 | 0.0005 | 5.0E-15 | -0.0660 | 0.0344 | 0.055 |
| rs6684154 | A | C | -0.0151 | 0.0016 | 1.4E-21 | 0.0851 | 0.1588 | 0.59 |
| rs66888782 | T | C | 0.0036 | 0.0007 | 2.5E-08 | 0.0089 | 0.0446 | 0.84 |
| rs6695321 | G | A | 0.0026 | 0.0004 | 3.4E-11 | 0.0183 | 0.0270 | 0.50 |
| rs67261871 | C | T | 0.0032 | 0.0004 | 1.4E-15 | -0.0117 | 0.0287 | 0.68 |
| rs6762589 | A | G | 0.0028 | 0.0004 | 2.1E-12 | -0.0192 | 0.0282 | 0.50 |
| rs682798 | G | A | -0.0025 | 0.0004 | 3.2E-10 | 0.0075 | 0.0272 | 0.78 |
| rs6872156 | G | A | 0.0026 | 0.0005 | 4.4E-08 | 0.0197 | 0.0345 | 0.57 |
| rs6879279 | C | G | -0.0042 | 0.0005 | 3.5E-15 | -0.0208 | 0.0352 | 0.56 |
| rs7001206 | A | G | -0.0026 | 0.0004 | 1.9E-09 | 0.0157 | 0.0318 | 0.62 |
| rs7018885 | C | T | -0.0038 | 0.0004 | 9.3E-18 | -0.0129 | 0.0338 | 0.70 |
| rs702484 | G | C | 0.0028 | 0.0004 | 1.8E-11 | -0.0252 | 0.0282 | 0.37 |
| rs7029757 | A | G | -0.0054 | 0.0007 | 9.6E-16 | -0.0510 | 0.0557 | 0.36 |
| rs7041363 | G | C | -0.0096 | 0.0004 | 1.4E-136 | -0.0061 | 0.0267 | 0.82 |
| rs7117339 | T | C | -0.0117 | 0.0006 | 1.8E-83 | 0.0128 | 0.0391 | 0.74 |
| rs7174367 | G | A | -0.0027 | 0.0004 | 2.3E-12 | -0.0361 | 0.0284 | 0.20 |
| rs7239575 | C | T | -0.0022 | 0.0004 | 1.1E-08 | 0.0140 | 0.0266 | 0.60 |
| rs7247349 | C | A | -0.0038 | 0.0004 | 2.7E-22 | -0.0275 | 0.0267 | 0.30 |
| rs72623176 | A | G | 0.0089 | 0.0010 | 9.8E-19 | -0.0118 | 0.0461 | 0.80 |
| rs72648770 | G | C | 0.0031 | 0.0005 | 1.4E-09 | -0.0324 | 0.0342 | 0.34 |
| rs72683923 | C | T | -0.0144 | 0.0014 | 3.2E-25 | 0.3374 | 0.1292 | 0.0090 |
| rs72787021 | C | G | 0.0029 | 0.0005 | 4.4E-09 | -0.0262 | 0.0331 | 0.43 |
| rs72801474 | A | G | -0.0047 | 0.0007 | 2.5E-13 | -0.0275 | 0.0568 | 0.63 |
| rs72959041 | A | G | 0.0074 | 0.0009 | 7.7E-16 | 0.0089 | 0.0554 | 0.87 |
| rs7306710 | C | T | 0.0022 | 0.0004 | 1.7E-08 | -0.0183 | 0.0267 | 0.49 |
| rs73087775 | T | C | 0.0025 | 0.0004 | 6.7E-09 | 0.0294 | 0.0362 | 0.42 |
| rs73613564 | T | C | -0.0026 | 0.0004 | 1.3E-10 | 0.0391 | 0.0278 | 0.16 |
| rs7402977 | A | G | -0.0026 | 0.0004 | 5.1E-09 | -0.0797 | 0.0298 | 0.0075 |
| rs7424120 | T | C | -0.0023 | 0.0004 | 4.9E-09 | -0.0232 | 0.0271 | 0.39 |
| rs75331444 | A | G | 0.0074 | 0.0008 | 3.0E-21 | 0.0141 | 0.0486 | 0.77 |
| rs75671243 | C | G | 0.0106 | 0.0011 | 1.2E-22 | -0.0133 | 0.0810 | 0.87 |
| rs7568960 | G | A | -0.0025 | 0.0004 | 1.2E-11 | -0.0029 | 0.0266 | 0.91 |
| rs76118307 | G | A | 0.0054 | 0.0009 | 8.8E-11 | -0.0946 | 0.0563 | 0.093 |
| rs76172548 | C | A | 0.0075 | 0.0011 | 3.6E-13 | -0.0116 | 0.0743 | 0.88 |
| rs7672435 | A | T | 0.0024 | 0.0004 | 4.0E-09 | 0.0014 | 0.0269 | 0.96 |
| rs76895963 | G | T | -0.0120 | 0.0015 | 4.1E-16 | 0.1115 | 0.0794 | 0.16 |
| rs77094191 | T | C | -0.0049 | 0.0008 | 3.0E-10 | 0.0648 | 0.0593 | 0.27 |
| rs7740107 | A | T | 0.0030 | 0.0004 | 4.9E-12 | 0.0051 | 0.0301 | 0.87 |
| rs77644333 | G | C | -0.0030 | 0.0005 | 1.5E-08 | 0.0279 | 0.0337 | 0.41 |
| rs7792 | T | G | -0.0028 | 0.0004 | 1.3E-12 | -0.0046 | 0.0270 | 0.87 |
| rs78173412 | T | C | -0.0052 | 0.0009 | 1.9E-09 | 0.0248 | 0.0594 | 0.68 |
| rs78376817 | C | T | 0.0042 | 0.0004 | 7.2E-22 | 0.0026 | 0.0316 | 0.94 |
| rs78843689 | T | A | -0.0045 | 0.0007 | 9.3E-10 | 0.0383 | 0.0436 | 0.38 |
| rs79124660 | A | G | -0.0061 | 0.0010 | 2.3E-10 | 0.0563 | 0.0680 | 0.41 |
| rs7913964 | G | A | 0.0023 | 0.0004 | 3.0E-08 | -0.0453 | 0.0277 | 0.10 |
| rs7916672 | T | C | 0.0022 | 0.0004 | 4.5E-08 | -0.0330 | 0.0268 | 0.22 |
| rs79287178 | A | G | -0.0180 | 0.0012 | 1.0E-55 | -0.1568 | 0.0673 | 0.020 |
| rs7966357 | G | C | -0.0029 | 0.0004 | 7.5E-13 | 0.0836 | 0.0288 | 0.0037 |
| rs7999034 | A | G | -0.0027 | 0.0004 | 7.6E-12 | -0.0033 | 0.0274 | 0.90 |
| rs8041057 | T | C | -0.0043 | 0.0004 | 1.4E-23 | -0.0738 | 0.0286 | 0.0098 |
| rs805047 | G | A | 0.0033 | 0.0004 | 5.3E-16 | 0.0181 | 0.0293 | 0.54 |
| rs8065221 | A | G | 0.0021 | 0.0004 | 4.9E-08 | -0.0587 | 0.0273 | 0.032 |
| rs8114057 | A | G | -0.0025 | 0.0004 | 2.0E-10 | 0.0098 | 0.0269 | 0.72 |
| rs819140 | G | A | -0.0034 | 0.0004 | 1.6E-19 | 0.0343 | 0.0269 | 0.20 |
| rs917116 | G | T | 0.0040 | 0.0005 | 6.1E-17 | -0.0268 | 0.0343 | 0.43 |
| rs9368188 | G | A | 0.0028 | 0.0004 | 2.5E-11 | -0.0257 | 0.0274 | 0.35 |
| rs9556403 | G | A | -0.0022 | 0.0004 | 3.0E-08 | -0.0951 | 0.0273 | 0.00050 |
| rs9583095 | T | C | 0.0024 | 0.0004 | 4.2E-09 | -0.0222 | 0.0281 | 0.43 |
| rs968468 | T | C | -0.0027 | 0.0004 | 2.2E-09 | 0.0165 | 0.0289 | 0.57 |
| rs9788910 | G | C | 0.0029 | 0.0004 | 1.6E-13 | -0.0420 | 0.0270 | 0.12 |
| rs9820178 | T | C | -0.0030 | 0.0005 | 2.5E-10 | -0.0208 | 0.0323 | 0.52 |
| rs9836030 | T | C | -0.0022 | 0.0004 | 3.0E-08 | -0.0299 | 0.0266 | 0.26 |
| rs9906971 | G | A | -0.0029 | 0.0005 | 1.0E-09 | 0.0627 | 0.0384 | 0.10 |
| Liver volume |  |  |  |  |  |  |  |  |
| rs1009064 | C | G | -0.0414 | 0.0073 | 1.3E-08 | 0.0177 | 0.0274 | 0.52 |
| rs10881959 | T | G | 0.0460 | 0.0071 | 6.8E-11 | -0.0307 | 0.0286 | 0.28 |
| rs1260326 | C | T | -0.0636 | 0.0071 | 5.4E-19 | 0.0653 | 0.0280 | 0.020 |
| rs139974673 | C | T | 0.2137 | 0.0227 | 4.9E-21 | -0.2589 | 0.1798 | 0.15 |
| rs193084249 | G | A | 0.1434 | 0.0232 | 6.5E-10 | -0.0393 | 0.0711 | 0.58 |
| rs4240624 | A | G | -0.1431 | 0.0122 | 1.3E-31 | 0.0739 | 0.0412 | 0.073 |
| rs58489806 | T | C | 0.0827 | 0.0124 | 3.0E-11 | -0.0393 | 0.0489 | 0.42 |
| rs62033400 | G | A | 0.0427 | 0.0072 | 2.9E-09 | 0.0118 | 0.0271 | 0.66 |
| rs79287178 | A | G | 0.1511 | 0.0211 | 8.0E-13 | -0.1568 | 0.0673 | 0.020 |
| rs9375447 | G | A | 0.0494 | 0.0070 | 1.2E-12 | 0.0289 | 0.0266 | 0.28 |

**Supplemental Figure 1.** Scatter plot of Mendelian randomization analyses for the causal relationship between several liver traits and CRC.

**
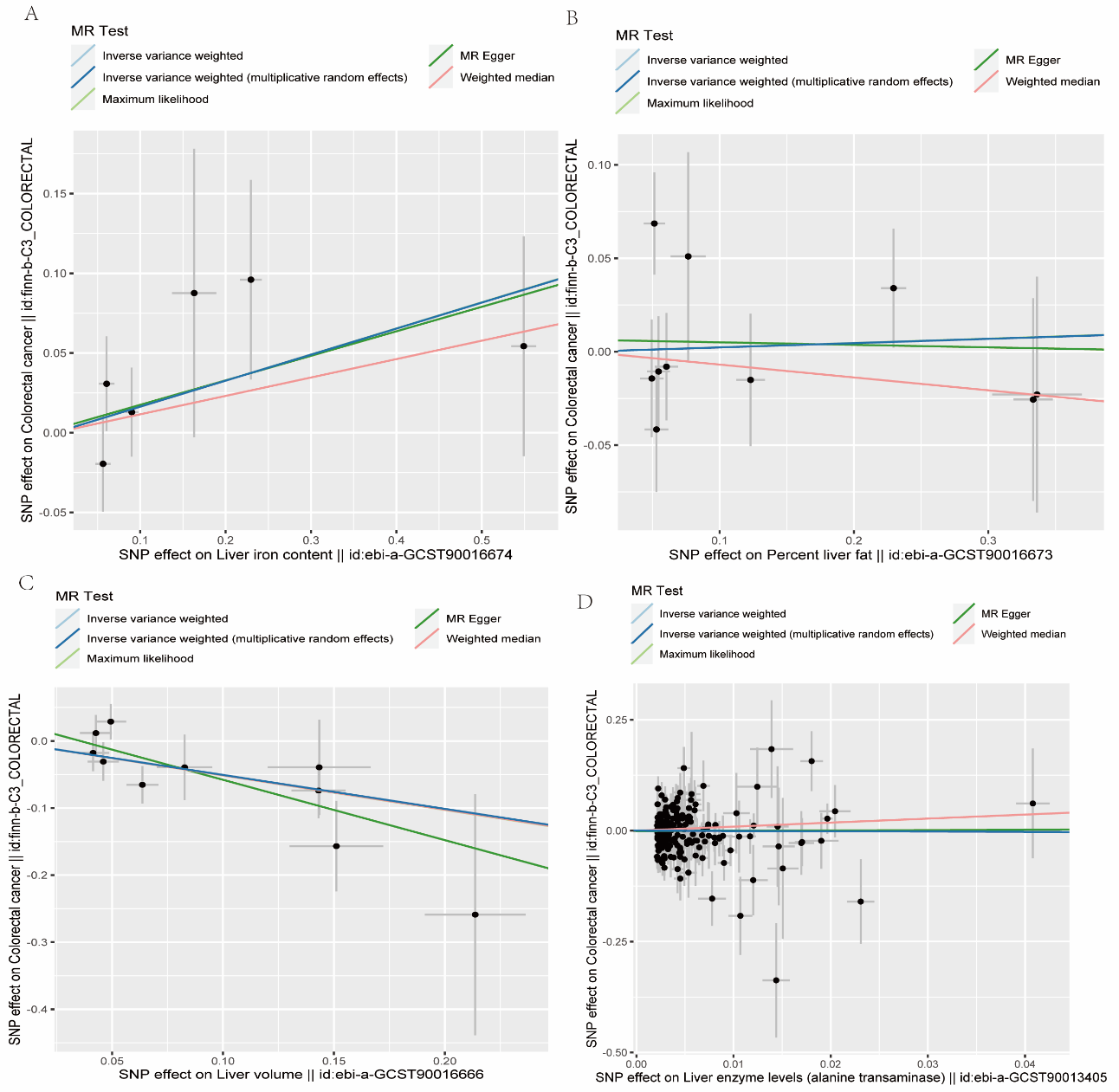
**

**Supplemental Figure 2.** The leave-one-out analysis to assess the influence of individual SNPs on the estimates. The results were consistent with estimates obtained when all available SNPs were included in the analysis.


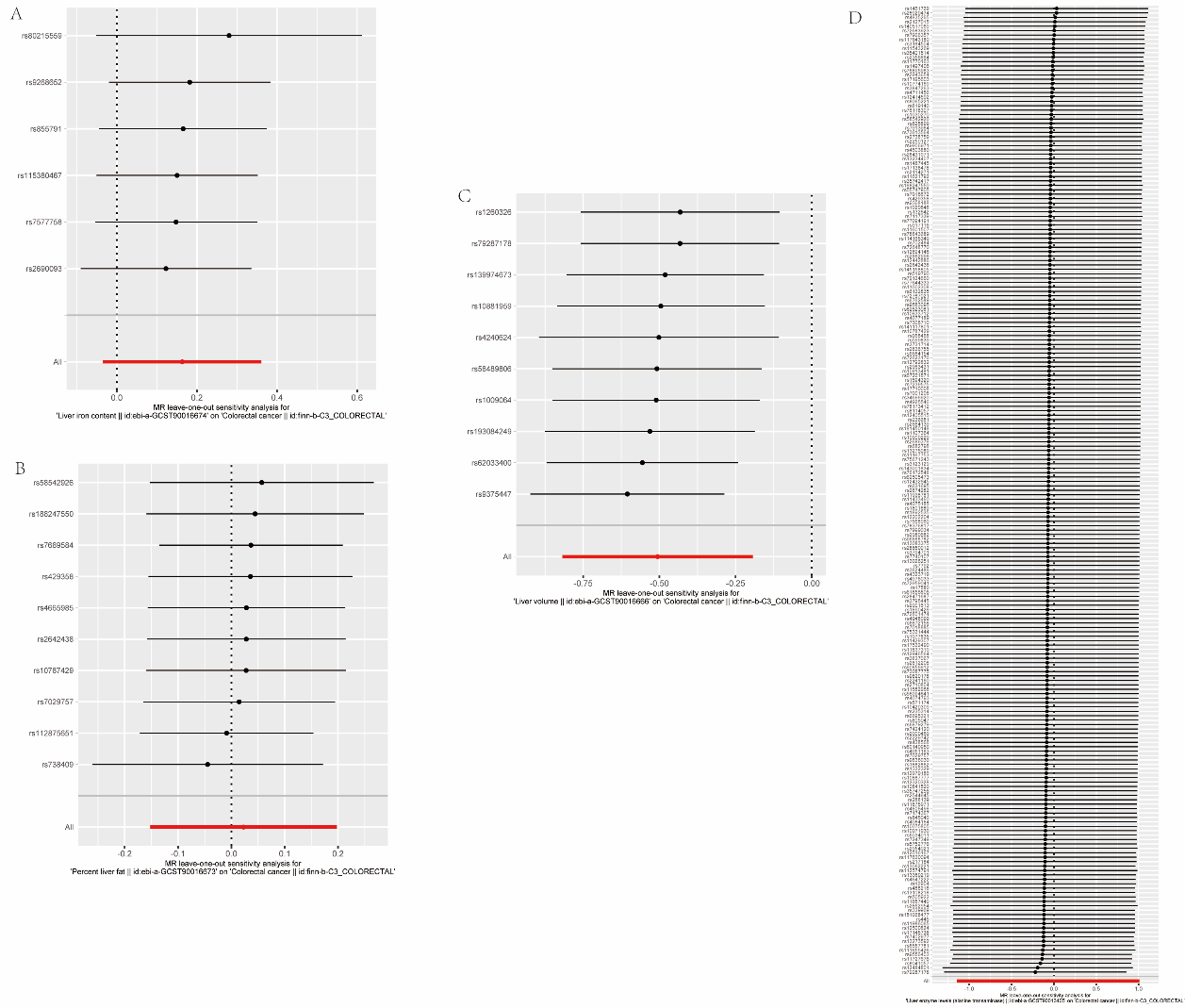


**Supplemental Figure 3.** Scatter plots generated from the IVW multivariable MR analysis.


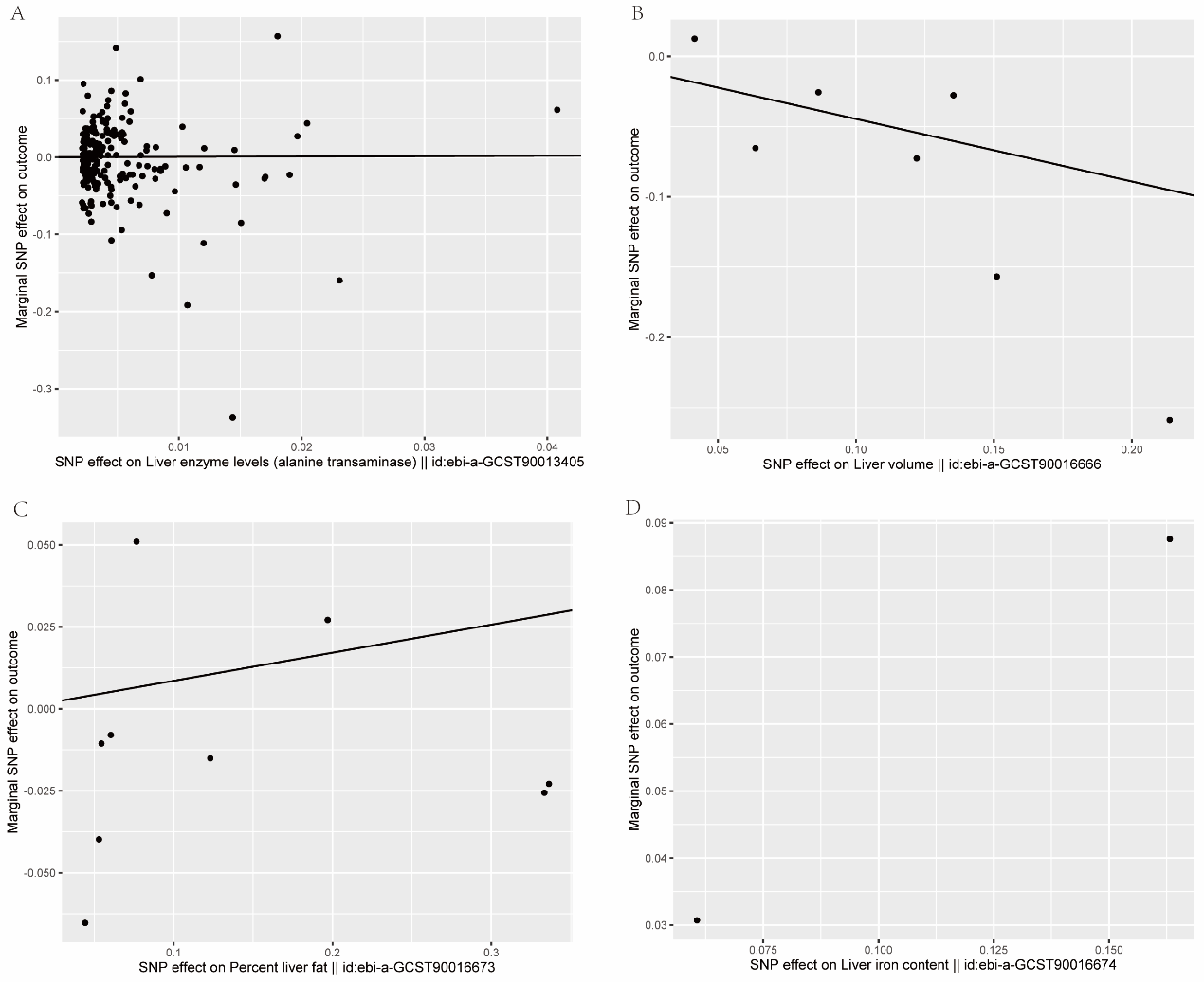


**Reference**

1. Liu Y, Basty N, Whitcher B, Bell JD, Sorokin EP, Van Bruggen N, et al. Genetic architecture of 11 organ traits derived from abdominal MRI using deep learning. eLife. 2021;10:e65554.

2. Pazoki R, Vujkovic M, Elliott J, Evangelou E, Gill D, Ghanbari M, et al. Genetic analysis in European ancestry individuals identifies 517 loci associated with liver enzymes. Nat Commun. 2021;12:2579.
